# Inflammation Markers in Patients with Bipolar Disorder Who Have Committed Offenses and Their Relationship with Criminal Behavior

**DOI:** 10.3390/medicina59101725

**Published:** 2023-09-27

**Authors:** Burcu Sırlıer Emir, Sevler Yıldız, Aslı Kazğan Kılıçaslan, Osman Kurt, Kerim Uğur, Muhammed Fatih Tabara, Süleyman Aydın

**Affiliations:** 1 Department of Psychiatry, Elazığ Fethi Sekin City Hospital, 23100 Elazığ, Turkey; dr_sevler@hotmail.com; 2 Department of Psychiatry, University of Bozok, 66300 Yozgat, Turkey; dr.kazgan@hotmail.com; 3 Department of Public Health, Adıyaman Provincial Health Directorate, 23100 Adıyaman, Turkey; drkurtosman@gmail.com; 4 Department of Psychiatry, University of Turgut Özal, 44900 Malatya, Turkey; premirek@gmail.com; 5 Department of Psychiatry, Elazig Mental Health and Diseases Hospital, 23100 Elazığ, Turkey; fatihtabara@gmail.com; 6 Department of Biochemistry, University of Fırat, 23119 Elazığ, Turkey; saydin1@firat.edu.tr

**Keywords:** bipolar disorder, inflammation, systemic inflammatory response index, systemic immune inflammatory index, high-density lipoprotein, criminal behavior

## Abstract

*Background and Objectives*: This study aimed to examine the function of various inflammation parameters and their interactions in the pathology of Bipolar disorder (BD) and to assess whether they could be biomarkers in the relationship between criminal behavior and BD. *Materials and Methods*: Overall, 1029 participants, including 343 patients with BD who have committed offenses, 343 nonoffending patients with BD, and 343 healthy controls, were included in this retrospective study. Neutrophil, lymphocyte, monocyte, and platelet counts; high-density lipoprotein (HDL-c) levels; systemic immune-inflammatory index (SII), systemic inflammatory response index (SIRI), neutrophil to high-density lipoprotein ratio (NHR), lymphocyte to high-density lipoprotein ratio (LHR), monocyte to high-density lipoprotein ratio (MHR), platelet to high-density lipoprotein ratio (PHR) were measured. *Results*: Significant differences were observed between the groups in terms of SII, SIRI, NHR, LHR, MHR, PHR, neutrophil, and monocyte values (*p* < 0.001). The lymphocyte counts were significantly higher in the patients with BD who committed offenses (*p* = 0.04). The platelet counts were significantly lower in the patients with BD who committed offenses compared to nonoffending patients with BD (*p* = 0.015). The HDL-c levels were significantly lower in the patients with BD who have committed offenses than those of nonoffending patients with BD (*p* < 0.001). Bipolar disorder, not receiving active psychiatric treatment, having a diagnosis of bipolar manic episodes, and having low platelet and HDL values constitute a risk of involvement in crime. *Conclusions*: The present study emphasizes the role of systemic inflammation in the pathophysiology of patients with BD with and without criminal offenses and the relationship between inflammation and criminal behavior.

## 1. Introduction

Bipolar disorder (BD) is a recurrent and chronic psychiatric disorder with an estimated lifetime prevalence of 2.4% [1,2]. Multifactorial causes, including genetic and environmental factors, have been implicated in the etiology of BD [3]. As in many psychiatric disorders, recent studies have drawn attention to the role of the dysregulation and inflammation of the immune system in the etiology of BD. In bipolar disorder, inflammation markers are involved in both disease stages and disease progression [4,5,6]. Levels of thiobarbuturic acid-reactive substances (TBARS) measured to evaluate oxidative stress were high in the acute stages of mania, hypomania, and depression. Siwek et al. reported that TBARS levels showed increased oxidative stress in the acute phase of bipolar disorder [7]. Microelements, including zinc and copper, which are involved in neuroinflammation [8], have been studied in mood disorders and copper levels were found to be low, while zinc levels were found to be higher in the control group [9]. The increase in peripheral inflammation marker levels and high incidence of some inflammatory and immune diseases in patients with BD support this hypothesis [10,11]. A meta-analysis reported that inflammatory cytokine levels were elevated not only during depressive and manic episodes but also in the euthymic periods [12]. Thus, BD is a disease with an inflammatory burden independent of its episodes.

Certain blood biomarkers have been employed to indicate inflammation; however, they are difficult and costly to analyze in routine practice, prompting the search for new and more accessible biomarkers. The neutrophil/lymphocyte ratio (NLR), monocyte/lymphocyte ratio (MLR), platelet/lymphocyte ratio (PLR), and platelet volume have been assessed as inflammatory markers in BD, with higher NLR, TLR, and MLR values in patients with BD undergoing a manic episode than in controls [13,14]. Furthermore, higher MLR and NLR values and monocyte and neutrophil counts were reported in patients with manic BD compared to those with depressive BD [15]. The systemic immune-inflammatory index (SII) is calculated using neutrophil, lymphocyte, and platelet counts and shows inflammation more comprehensively. It has been used in various studies as an indicator of inflammation and prognosis [16,17]. SII can reportedly predict poor prognosis better in some cancers than NLR and PLR [18]. Neutrophil, monocyte, and lymphocyte counts, which are effective for determining the relationship between inflammation and immune response, have been used to calculate the systemic inflammatory response index (SIRI). Reportedly, SIRI can be used as an indicator of poor prognosis in cancer, and it better predicts the benefit of treatment [19,20]. These two indices reflect the interaction between inflammation, thrombocytosis, and the immune response.

High-density lipoprotein (HDL-c) exhibits important effects during the acute phase of inflammation, such as reducing the inflammatory response in monocytes and platelet aggregation. In addition to its anti-inflammatory effect, it demonstrates antithrombotic and antioxidant effects [21]. Furthermore, decreases in HDL-c, which affects the central nervous system, can reportedly increase the incidence of affective disorders [22]. Another study revealed that HDL-c levels were lower in the first-degree relatives of patients with BD, suggesting a possible relationship between HDL-c levels and BD [23].

Patients with bipolar disorder are at risk of harming themselves and others, especially during episodes [24]. It has been shown that the suicide probability of psychiatric patients can be predicted by hemogram parameters [25]. Patients with BD often exhibit criminal behavior during the manic period because of increased energy and grandiosity and during the depressive period owing to psychotic symptoms [26]. In a study comparing patients who have committed offenses with nonoffending patients, neutrophil and monocyte counts were higher in the former compared to the latter. In contrast, the NLR, PLR, and MLR values were lower in both patient groups than in healthy controls. These parameters were associated with indicators of inflammation in patients with BD who have committed offenses [27].

The involvement of neutrophils, monocytes, lymphocytes, platelets, and HDL-c in inflammation and the interaction of these parameters with each other have attracted the attention of researchers as a new biomarker of inflammation. Indeed, based on this hypothesis, these biomarkers effectively determine the prognosis of and predict disease in cancer, Parkinson’s disease, and erectile dysfunction [20,28,29]. Studies have been conducted on hemogram and biochemistry parameters to indicate inflammation in patients with bipolar disorder. It has been shown that increased PLR levels in patients with bipolar disorder may be associated with (hypo)mania [30]. It has also been said that differences in the concentrations of blood cholesterol levels in patients with bipolar disorder will be associated with the onset of mood attacks [31]. This study aimed to determine SII, SIRI, NHR, LHR, MHR, and PHR values and examine their usability as biomarkers for patients with BD and the relationship of BD with criminal behavior.

## 2. Materials and Methods

### 2.1. Sample

The current study was approved by the Research Ethics Board (decision no: 2022/14-15) and conducted in accordance with the Helsinki Declaration (2013). This study was conducted retrospectively, and patient information was accessed through the electronic registry system. Patients between the ages of 18 and 65 were diagnosed with BD according to the Diagnostic and Statistical Manual of Mental Disorders, Fifth Edition (DSM-5) criteria between January 2021 and January 2023. Age- and gender-matched healthy controls were included in the study. As our female forensic psychiatry ward is inactive, the study sample comprised only men. The concept of crime has violent and non-violent criminal behavior. Violent crime is aimed at harming the physical integrity of the person crimes. Murder, injury, and sexual assault are considered in this category. Crimes that do not harm the physical integrity of the person, such as insults, threats, and theft, are non-violent [32]. The first group consisted of BD patients brought to Elazığ Fethi Sekin City Hospital High-Security Forensic Psychiatry (HSFAP) Clinic by law enforcement officers after committing a crime and were hospitalized. The second group comprised nonoffending patients with BD who were outpatients, and the third group comprised healthy male controls without any psychiatric diagnosis. The exclusion criteria included the presence of other medical diseases that may affect inflammation (acute or chronic endocrinologic, inflammatory, infectious, or autoimmune diseases), active infection, steroid or similar immunomodulatory drug use, organic brain pathology, mental retardation, history of alcohol/substance abuse in the last six months, and additional diagnosed mental illnesses.

### 2.2. Data Collection Tools

A sociodemographic and clinical data form prepared by the researchers based on clinical experience and according to the relevant literature was used to obtain data. In addition to demographic information such as age and marital status, the demographic and clinical evaluation form also includes clinical evaluation questions such as the crime committed by the participants and their treatment history in the forensic psychiatry service.

### 2.3. Laboratory Samples

Blood samples were routinely collected at 07.00 in the morning when the patients were fasting, and venous blood samples were obtained using the antecubital vein. Complete blood count was analyzed using DXH-800 (Beckman Coulter, Inc, Miami, FL, USA), and biochemical parameters were analyzed via the Beckman AU-5800 device (Beckman Coulter Diagnostics, Indianapolis, IN, USA). Neutrophil, lymphocyte, monocyte, and platelet counts, and HDL-c levels were measured in the patient and control groups. The NHR, PHR, and MHR were calculated manually (neutrophil count/HDL-c, platelet count/HDL-c, and monocyte count/HDL-c, respectively). SIRI and SII were obtained using the formulas SIRI = neutrophil count × monocyte/lymphocyte count and SII = platelet count × monocyte/lymphocyte count.

### 2.4. Statistical Analysis

The data obtained were evaluated using the SPSS (Statistical Package for Social Sciences; SPSS Inc., Chicago, IL, USA) 22 package program. Descriptive statistics were presented as numbers, categorical variables as percentages, and continuous variables as mean ± standard deviation (mean ± SD). Chi-square analysis (Pearson Chi-square) was used to compare categorical variables between the groups. The conformity of continuous variables to normal distribution was evaluated via Kolmogorov–Smirnov test. Kruskal–Wallis’s test was used to compare more than two variables. Pairwise comparisons were made for those with significant differences in the Kruskal-Wallis’s test, and Bonferroni correction was made. Logistic regression analysis was used to calculate the risk of involvement in crime. Those significant in pairwise comparisons were included in the model for multivariate. The statistical significance level was accepted as a *p*-value of <0.05 in all the analyses.

## 3. Results

A total of 1029 participants, including 343 patients with BD who have committed offenses, 343 nonoffending patients with BD, and 343 healthy controls, were included in the study. All patients were male, and there was no significant difference between the groups in terms of age (*p* = 0.305). The percentage of patients undergoing active psychiatric treatment in the BD group that have committed offenses (60.6%) was significantly lower compared to that in the nonoffending BD group (86.6%) (*p* < 0.001). While 93% of the patients with BD who have committed offenses and 92.72% of nonoffending patients with BD had a history of psychiatric illness, none of the healthy controls did. There was a significant difference between the groups in terms of having a history of psychiatric illness (*p* < 0.001). The rate of bipolar depression in the patients with BD who have committed offenses (21.9%) was significantly lower compared to that in nonoffending patients with BD (31.2%) (*p* = 0.003). While 31.2% of patients with BD who have committed offenses committed violent crimes, 68.8% committed nonviolent crimes. Furthermore, 64.1% of the patients with BD who have committed offenses received treatment once, and 35.9% received treatment twice in the high-security forensic psychiatry service ward. While 86.6% of the patients with BD who have committed offenses received psychiatric treatment outside the YGAP, this rate was 86.9% for nonoffending patients with BD, and no significant difference was found between the groups (*p* = 0.910) (Table 1).

Significant differences were observed between the groups in terms of SII, SIRI, NHR, LHR, MHR, PHR, neutrophil, and monocyte values (*p* < 0.001). All values were significantly lower in the patients with BD who have committed offenses compared to that in the nonoffending patients with BD and healthy controls. The lymphocyte count was significantly higher in the patients with BD who have committed offenses than in healthy controls (*p* = 0.04). The platelet count was significantly lower in the patients with BD who have committed offenses than the nonoffending patients with BD (*p* = 0.015). Lastly, HDL-c level was significantly lower in the patients with BD who have committed offenses compared to that in the nonoffending patients with BD and healthy controls (*p* < 0.001) (Table 2).

The SIRI value of those with a previous history of psychiatric illness was significantly higher than those with no previous psychiatric illness (*p* = 0.024). The SII value was significantly higher in patients who received inpatient psychiatric treatment outside of high-security forensic psychiatry services than those who did not (*p* = 0.022). When the crime content was divided into violent and non-violent and compared in terms of SII, SIRI, NHR, LHR, MHR, and PHR parameters, no significant difference was observed between violent and non-violent crime. (Table 3).

In the logistic regression analysis performed to calculate the risk of committing crime in patients with bipolar disorder, not receiving active psychiatric treatment, having a diagnosis of bipolar manic episode, bipolar manic + depression episode, and having low platelet and HDL values constitute a risk in terms of involvement in crime (Table 4).

## 4. Discussion

SII and SIRI are new biomarkers studied to predict the prognosis of numerous neurological and cardiac diseases, especially in cancers [16,17,18,19,20,30,31,32,33]. The fact that inflammation is a pathological mechanism in psychiatric diseases has led researchers to investigate these two biomarkers [34]. Knowledge regarding the neurobiology of BD is rapidly expanding, and numerous studies have reported that inflammatory processes play an essential role in the pathophysiology of BD [35,36]. Although not all patients exhibit markedly elevated inflammation marker levels, there is evidence that abnormal immune activity occurs in all the stages of the disease, which can probably explain the high incidence of comorbid inflammatory diseases in this patient population [37,38]. Herein, we examined several parameters in patients with BD who have committed offenses, nonoffending patients with BD, and healthy controls. We found that SII, SIRI, NHR, LHR, MHR, and PHR values and neutrophil, lymphocyte and monocyte counts were significantly higher in the patients with BD who have committed offenses than in the other two groups. Conversely, platelet count, and HDL-c levels were significantly lower in the patients with BD who have committed offenses compared to the nonoffending patients and healthy controls.

Neutrophil, monocyte, lymphocyte, platelet, and HDL-c values are closely associated with inflammation and oxidative stress [39]. Neutrophils are key cell types in the innate immune system and are the most common type of white blood cell in the body. Lymphocytes are components of the adaptive immune system and play an important role in the body’s immune response, including antibody production and cell-mediated immunity. Monocytes are vital to the innate immune response and play an important role in the secretion of proinflammatory and pro-oxidant cytokines. Platelets regulate the permeability of endothelial cells, with activated platelets exhibiting inflammatory functions. HDL-c exerts pleiotropic protective functions, including anti-inflammatory, antioxidant, antithrombotic, and immunomodulatory properties. HDL-c inhibits the activation, binding, diffusion, and migration of neutrophils and the activity of monocytes.

Furthermore, HDL-c may inhibit T-cell activation by disrupting lipid release [40,41,42]. Considering this extensive interaction of HDL-c with other cells, the combined biomarkers of NHR, LHR, MHR, and PHR, which include HDL-c, may be better at indicating inflammation. Xu et al. [43] reported that the MHR and NHR values were significantly lower in these patients than in healthy controls; however, no difference was detected in the LHR and PHR values. Wei et al. [39] stated that MHR and NHR can distinguish patients with BD undergoing a manic episode from healthy controls, while MHR can distinguish patients with BD undergoing a depressive episode from healthy controls. In addition, previous studies have also reported that MHR was significantly higher in patients with schizophrenia than in healthy controls [44]. Herein, the NHR, LHR, MHR, and PHR values were significantly higher in the patients with BD who have committed offenses compared to nonoffending patients and healthy controls. This may be related to the degree or severity of inflammation in the patients with BD who have committed offenses. Again, the type of criminal offense influences inflammation markers [27]. Inflammation parameters were higher in homicide, one of the violent crimes, than in other crimes [45].

SII is a biomarker of systemic inflammation that can be easily calculated using neutrophil, lymphocyte, and platelet counts. Previous studies emphasized that SII is important in predicting the prognosis of various physical diseases such as tumors, cerebral infarction, cardiovascular diseases, and acute pancreatitis [16,17,20,31,33]. Wei and Xu reported that the SII values of patients diagnosed with BD were higher compared to those of healthy controls; moreover, SII values were significantly higher in patients with BD undergoing a manic episode compared to those undergoing a depressive episode [39,43]. Dadouli reported that although SII was higher in patients with BD, no significant difference was found between those undergoing a manic or depressive episode [46]. In a study in which patients with BD undergoing a manic episode were compared to healthy controls, SII was significantly higher in the patient group than in the healthy controls [47]. Herein, SII was significantly higher in the patients with BD who have committed offenses compared to nonoffending patients with BD and healthy control. Although SII was higher in the nonoffending patients with BD compared to the healthy controls, the difference was not statistically significant. This may be because of the lower treatment compliance of the offending patient group. The relationship between the SII values and the prognosis and severity of the disease can be investigated in future studies.

SIRI is a new biomarker of inflammation based on neutrophil, lymphocyte, and monocyte counts. Like SII, SIRI has been extensively studied in physical diseases such as tumors, cardiovascular diseases, and stroke [18,19,20]. However, there are limited studies examining SIRI values in patients with BD. Wei et al. reported that SIRI was significantly higher in patients with schizophrenia and BD than healthy controls. The highest SIRI value was obtained in the manic BD group, followed by the schizophrenia and depressive BD groups. The authors also emphasized that a high SIRI value was an independent biomarker to distinguish patients who were manic from healthy controls [38]. Xu et al. [43] also reported that SIRI was significantly higher in patients with BD compared to healthy controls, regardless of whether manic or depressed. Korkmaz et al. [48] compared patients with BD in acute mania and early remission with healthy controls and found that the SIRI value was the highest in patients undergoing acute mania. Although the SIRI value decreased in the early remission period of the patients, it remained significantly higher than that of the healthy controls. Herein, we found that the SIRI values of the patients with BD who have committed offenses were significantly higher than those of healthy controls and nonoffending patients with BD. Therefore, although our results indicate that SIRI can be used as a biomarker in patients with BD, further studies are needed.

We found that in patients with bipolar disorder, not receiving active psychiatric treatment, being in the manic period of the disease, and having low platelet and HDL values among blood parameters constitute a risk for involvement in crime. Fazel et al. reported that patients in the manic period were more prone to violence [49] and that regular drug use in this patient group reduced crime rates [50]. Since cholesterol has been associated with impulsivity as a moderator of serotonergic function, although some studies have reported that HDL level is associated with crime [51]. While we did not detect any difference in platelet counts between those who had committed a crime and those who had not [27], we observed that low levels of platelets increased the tendency towards crime.

Our limitation of this study was its retrospective design and only male recruitment. It is known that psychiatric medications can affect inflammation markers. One of our limitations was that some of the patients with bipolar disorder were receiving treatment [52]. Although it is known that criminal behavior may vary according to socioeconomic status, educational level and ethnic origin [53,54], we could not evaluate these parameters because our study was retrospective.

## 5. Conclusions

In conclusion, as demonstrated in our study and previously published studies, SII and SIRI values, which are biomarkers of inflammation, are significantly higher in patients with BD. The fact that they are highest during mania and decrease slightly in remission suggests that they can be used to predict the prognosis and severity of the disease. Our study is the first study evaluating patients diagnosed with BD as involved in crime and not involved in crime. We observed that inflammation parameters may affect the severity and prognosis of the disease in patients with bipolar disorder. In addition, since patients receiving active psychiatric treatment may affect the tendency to commit crime and even the type of crime committed, we believe that early detection of this condition and effective treatment may reduce crime rates. Our findings will shed light on future prospective studies in patients with bipolar disorder.

## Figures and Tables

**Table 1:** Comparison of all characteristics by groups.

		Patients with BD Who Have Committed Offenses	Nonoffending Patients with BD	Control	*p*
		*n* (%)	*n* (%)	*n* (%)	
Age, Mean ± SD		36.45 ± 10.77	37.30 ± 11.15	36.55 ± 12.39	0.305 *
Active psychiatric treatment	Yes	208 (60.6)	297 (86.6)	-	<0.001 **
	No	135 (39.4)	46 (13.4)		
Previous history of psychiatric illness	Yes	319 (93.0)	318 (92.7)	0 (0.0)	<0.001 **
	No	24 (7.0)	25 (7.3)	343 (100)	
Diagnosis	Bipolar manic episode	71 (20.7)	41 (12.0)	-	0.003 **
	Bipolar depression episode	75 (21.9)	107 (31.2)		
	Bipolar manic+depression episode	51 (14.9)	51 (14.9)		
	Bipolar remission	146 (42.6)	144 (42.0)		
Duration illness, mean ± SD, year	9.6 ± 7.15		9.3 ± 8.32	-	-
Criminal offense type	Violent	107 (31.2)	-	-	-
	Nonviolent	236 (68.8)			
Number of treatments in High-Security Forensic Psychiatry (HSFP) Clinic	1	220 (64.1)	-	-	-
	≥2	123 (35.9)			
Inpatient psychiatric treatment outside the High-Security Forensic Psychiatry (HSFP) Clinic	Yes	297 (86.6)	298 (86.9)	-	0.910 **
	No.	46 (13.4)	45 (13.1)		

* Kruskal–Wallis test, ** Chi-square test.

**Table 2:** Comparison of laboratory parameters according to groups.

	Patients with BD Who Have Committed Offenses	Nonoffending Patients with BD	Control	*p* *
	Mean ± SD	Mean ± SD	Mean ± SD	
Systemic immune inflammatory index (SII)	748.94 ± 632.73 ^a^	564.57 ± 332.24 ^b^	551.64 ± 308.78 ^b^	**<0.001**
Systemic inflammatory response index (SIRI)	2.09 ± 2.79 ^a^	1.19 ± 0.78 ^b^	1.32 ± 1.15 ^b^	**<0.001**
Neutrophil to high-density lipoprotein ratio (NHR)	0.13 ± 0.06 ^a^	0.09 ± 0.04 ^b^	0.09 ± 0.04 ^b^	**<0.001**
Lymphocyte to high-density lipoprotein ratio (LHR)	0.06 ± 0.02 ^a^	0.05 ± 0.02 ^b^	0.04 ± 0.02 ^b^	**<0.001**
Monocyte to high-density lipoprotein ratio (MHR)	0.02 ± 0.01 ^a^	0.01 ± 0.01 ^b^	0.01 ± 0.01 ^b^	**<0.001**
Platelet to high-density lipoprotein ratio (PHR)	6.15 ± 1.83 ^a^	5.69 ± 1.88 ^b^	5.42 ± 1.86 ^b^	**<0.001**
Neutrophil	5.50 ± 2.06 ^a^	4.27 ± 1.55 ^b^	3.99 ± 1.26 ^b^	**<0.001**
Lymphocyte	2.26 ± 0.85 ^a^	2.19 ± 0.68 ^a,b^	2.09 ± 0.72 ^b^	**0.04**
Monocyte	0.66 ± 0.24 ^a^	0.56 ± 0.22 ^b^	0.57 ± 0.22 ^b^	**<0.001**
Platelet	252.21 ± 63.61 ^a^	264.86 ± 65.18 ^b^	258.93 ± 58.88 ^a.b^	**0.015**
High-density lipoprotein (HDL-c)	42.43 ± 8.40 ^a^	48.66 ± 11.97 ^b^	50.09 ± 11.90 ^b^	**<0.001**

* Kruskal–Wallis’s test, ^a^,^b^ = group where the difference originates.

**Table 3:** Comparison of SII, SIRI, NHR, LHR, MHR, MHR, and PHR values in patients with BD who have committed offenses and nonoffending patients with respect to various parameters.

		SII	SIRI	NHR	LHR	MHR	PHR
		Mean ± SD	Mean ± SD	Mean ± SD	Mean ± SD	Mean ± SD	Mean ± SD
Active psychiatric treatment	Yes	718.53 ± 541.99	2.02 ± 2.45	0.13 ± 0.06	0.06 ± 0.02	0.02 ± 0.01	6.06 ± 1.82
	No	795.80 ± 751.24	2.19 ± 3.25	0.14 ± 0.06	0.06 ± 0.03	0.02 ± 0.01	6.28 ± 1.83
*p* *		0.579	0.871	0.495	0.703	0.785	0.297
Previous history of psychiatric illness	Yes	762.34 ± 647.46	2.14 ± 2.87	0.13 ± 0.06	0.06 ± 0.02	0.02 ± 0.01	6.17 ± 1.85
	No	570.81 ± 348.51	1.39 ± 1.05	0.13 ± 0.05	0.06 ± 0.03	0.01 ± 0.01	5.85 ± 1.48
*p* *		0.08	**0.024**	0.680	0.381	0.195	0.611
Diagnosis	Bipolar manic episode	697.05 ± 591.59	1.89 ± 3.32	0.13 ± 0.05	0.06 ± 0.02	0.02 ± 0.01	6.31 ± 1.86
	Bipolar depressive episode	754.74 ± 523.55	2.15 ± 1.90	0.13 ± 0.05	0.05 ± 0.02	0.02 ± 0.01	6.01 ± 1.80
	Bipolar manic+depression episode	760.54 ± 936.84	2.30 ± 4.63	0.13 ± 0.06	0.05 ± 0.03	0.02 ± 0.01	6.21 ± 1.89
	Bipolar remission	767.14 ± 575.39	2.08 ± 1.93	0.14 ± 0.06	0.06 ± 0.03	0.02 ± 0.01	6.11 ± 1.81
*p* **		0.480	0.089	0.691	0.488	0.480	0.731
Type of crime	Violent	744.23 ± 575.56	1.99 ± 1.85	0.13 ± 0.05	0.06 ± 0.02	0.02 ± 0.01	6.27 ± 1.76
	Nonviolent	751.07 ± 658.18	2.13 ± 3.12	0.13 ± 0.06	0.05 ± 0.03	0.02 ± 0.01	6.09 ± 1.86
*p* *		0.984	0.680	0.584	0.180	0.111	0.346
Number of treatments in High-Security Forensic Psychiatry (HSFP) Clinic	1	685.35 ± 402.19	1.79 ± 1.34	0.13 ± 0.05	0.06 ± 0.02	0.02 ± 0.01	6.08 ± 1.74
	≥2	862.67 ± 900.89	2.62 ± 4.26	0.14 ± 0.06	0.06 ± 0.03	0.02 ± 0.01	6.27 ± 1.97
*p* *		0.367	0.208	0.941	0.944	0.474	0.417
Inpatient psychiatric treatment outside the High-Security Forensic Psychiatry (HSFP) Clinic	Yes	775.97 ± 663.89	2.16 ± 2.95	0.14 ± 0.06	0.05 ± 0.02	0.02 ± 0.01	6.18 ± 1.86
	No	574.44 ± 327.98	1.61 ± 1.18	0.13 ± 0.05	0.06 ± 0.03	0.02 ± 0.01	5.95 ± 1.59
*p* *		**0.022**	0.111	0.426	0.103	0.953	0.539

* Mann-Whitney U test, ** Kruskal–Wallis test.

**Table 4:** Logistic regression analysis of the presence of criminal involvement.

	B	S.E.	*p*	OR	95% C.I. for OR	
					Lower	Upper
Active psychiatric treatment (reference = yes)	1.403	0.229	**<0.001**	4.065	2.596	6.367
Previous history of psychiatric illness (reference = no)	0.612	0.388	0.115	1.845	0.862	3.950
Diagnosis (Bipolar depressive episode)						
Diagnosis (Bipolar manic episode)	1.043	0.302	**0.001**	2.839	1.571	5.130
Diagnosis (Bipolar manic+depression episode)	0.597	0.298	**0.045**	1.817	1.014	3.257
Diagnosis (Bipolar remission)	0.374	0.229	0.102	1.454	0.928	2.277
Inpatient psychiatric treatment outside High-Security Forensic Psychiatry (HSFP) Clinic (reference = no)	0.539	0.299	0.072	1.714	0.954	3.081
Systemic immune inflammatory index (SII)	0.001	0.001	0.467	1.001	0.999	1.003
Systemic inflammatory response index (SIRI)	0.475	0.292	0.104	1.608	0.907	2.852
Neutrophil to high-density lipoprotein ratio (NHR)	−13.298	12.232	0.277	0.000	0.000	43,335
Lymphocyte to high-density lipoprotein ratio (LHR)	−7.402	21.667	0.733	0.001	0.000	16,922,618
Monocyte to high-density lipoprotein ratio (MHR)	−27.640	43.727	0.527	0.000	0.000	16,453,112
Platelet to high-density lipoprotein ratio (PHR)	0.402	0.267	0.132	1.495	0.886	2.523
Neutrophil	0.527	0.313	0.092	1.693	0.917	3.126
Lymphocyte	0.665	0.579	0.250	1.945	0.626	6.047
Monocyte	0.434	1.146	0.705	1.544	0.163	14.580
Platelet	−0.017	0.007	**0.009**	0.983	0.970	0.996
High-density lipoprotein (HDL-c)	−0.050	0.020	**0.012**	0.951	0.915	0.989

B: regression coefficient, S.E.: standard error, *p*: Statistical significance, OR: Odds ratio, C.I.: Confidence interval.

## Data Availability

Data are available upon reasonable request.
